# G protein-coupled receptors as new therapeutic targets for type 2 diabetes

**DOI:** 10.1007/s00125-015-3825-z

**Published:** 2015-12-12

**Authors:** Frank Reimann, Fiona M. Gribble

**Affiliations:** Wellcome Trust-MRC Institute of Metabolic Science, Metabolic Research Laboratories, University of Cambridge, Addenbrooke’s Hospital Box 289, Hills Road, Cambridge, CB2 0QQ UK

**Keywords:** Diabetes, G protein-coupled receptor, GIP, GLP-1, Glucagon, Insulin, Obesity

## Abstract

G protein-coupled receptors (GPCRs) in the gut–brain–pancreatic axis are key players in the postprandial control of metabolism and food intake. A number of intestinally located receptors have been implicated in the chemo-detection of ingested nutrients, and in the pancreatic islets and nervous system GPCRs play essential roles in the detection of many hormones and neurotransmitters. Because of the diversity, cell-specific expression and ‘druggability’ of the GPCR superfamily, these receptors are popular targets for therapeutic development. This review will outline current and potential future approaches to develop GPCR agonists for the treatment of type 2 diabetes. This review summarises a presentation given at the ‘Novel approaches to treating type 2 diabetes’ symposium at the 2015 annual meeting of the EASD. It is accompanied by a commentary by the Session Chair, Michael Nauck (DOI: 10.1007/s00125-015-3823-1).

Therapeutics that promote insulin secretion have been a mainstay of type 2 diabetes treatment for many years. However, with the rising impact of obesity on the incidence of type 2 diabetes comes an increasing need to target body weight as well as blood glucose control. Recent years have witnessed an increasing interest in the gut endocrine system as a source of novel peptides that regulate energy balance. Glucagon-like peptide 1 (GLP-1) is a prime example, and has been exploited pharmacologically for its ability both to trigger glucose-dependent insulin secretion and to reduce appetite [1]. The GLP-1 receptor is just one of a range of G protein-coupled receptors (GPCRs) that enable drugs to target tissues of relevance for type 2 diabetes and obesity, and ligands to other GPCRs, including free fatty acid receptor 1 (FFAR1, also known as GPR40), GPR119 and the glucose-dependent insulinotropic polypeptide (GIP) receptor, have progressed recently through to clinical trials in humans (for examples, see [2, 3]).

## Why GPCRs?

The GPCR superfamily is comprised of receptors involved in the detection of a wide range of chemicals, including nutrients, hormones, neurotransmitters, odorants and tastants [4]. Their specialised ligand-binding domains are tuned so that each receptor responds only to a narrow range of chemical structures. In turn, this provides unique sites for high affinity and specific drug binding. Restricted expression of individual GPCRs to a limited range of target tissues is a mechanism widely used by the body to enable highly specific inter-organ crosstalk, with the endocrine system being a prime example. New GPCR-based drug therapies exploit the cell specificity of receptor expression to achieve activation of a selected range of target tissues, including pancreatic beta cells, gut endocrine cells and neuronal populations involved in the suppression of appetite (for examples, see [1, 3, 5]). They aim to fool the body into believing that it has just eaten a meal, thereby enhancing glucose-dependent insulin secretion and reducing hunger.

GPCRs that have received recent attention in the field of diabetes therapeutics include the incretin receptors (GLP1R, GIPR), GPR119, FFAR1 (GPR40), FFAR4 (GPR120) and the bile acid receptor GPBAR1 (TGR5) (for examples, see [2, 3, 5–7]) (see text box). Activation of these receptors is normally associated with the postprandial state, as they are variously the targets for nutrients, bile acids and gut hormones [4]. Although there is a well-recognised GPCR capable of detecting glucose—the sweet taste receptor TAS1R2/3 heterodimer [8]—non-GPCR mechanisms appear to dominate postprandial glucose-dependent insulin and GLP-1 release, which are instead achieved through metabolism-dependent closure of ATP-sensitive potassium (K_ATP_) channels in beta cells or electrogenic sodium-dependent glucose transport in GLP-1-secreting L cells [9, 10].

**Table:** 

**Summary of the natural ligands, targets and actions of example GPCRs located in intestinal and pancreatic endocrine cells**			
Receptor	Natural ligands	Target cell types/tissues	Effect of GPCR agonism
GLP1R	GLP-1, oxyntomodulin	Pancreatic beta and delta cells, brain, vagus nerve	↑ insulin, ↓ glucagon, ↓ gastric emptying
GIPR	GIP	Pancreatic alpha, beta and delta cells, adipose tissue	↑ insulin, ↑ glucagon, ↑ fat storage
GCGR	Glucagon, oxyntomodulin	Pancreatic beta cells, liver, adipose tissue, brain	↑ insulin, ↑ energy expenditure, ↓ food intake
GPR119	Oleoylethanolamide, monooleoylglycerol	Enteroendocrine cells, pancreatic alpha and beta cells	↑ GLP-1, ↑ GIP, ↑ insulin (possible ↑ glucagon)
FFAR1 (GPR40)	Long-chain NEFA	Enteroendocrine cells, pancreatic beta cells	↑ GLP-1, ↑ GIP, ↑ insulin
FFAR4 (GPR120)	Long-chain NEFA	Enteroendocrine cells, pancreatic delta cells	↑ GLP-1, ↑ GIP, ↓ somatostatin
GPBAR1 (TGR5)	Bile acids	Enteroendocrine cells, adipose tissue	↑ GLP-1, ↑ energy expenditure

## The GLP-1 receptor as an exemplary beta cell GPCR

The GLP-1 receptor GLP1R is probably the best characterised GPCR to have been harnessed to date for the treatment of diabetes. It is targeted by injectable GLP-1 mimetics as well as by raised circulating active GLP-1 concentrations achieved following dipeptidyl peptidase 4 (DPP4) inhibition, but has proved a difficult target for developing small molecule ligands that could be administered in oral formulations [11]. Together with the receptor for GIP, GLP1R activation underlies the physiological incretin effect, responsible for about 50% of postprandial insulin secretion [12]. The incretin effect relies on the high expression by pancreatic beta cells of GLP1R and GIPR, activation of which results in recruitment of G_αs_ G protein subunits and elevation of cytoplasmic cAMP concentrations [13].

Activation of GLP1R has the highly favourable property that it normally only triggers insulin secretion in the context of a raised blood glucose concentration. Hypoglycaemic side effects of therapeutic GLP-1 mimetics are therefore rare unless used in combination with sulfonylureas [14]. This is because cAMP is a poor beta cell secretagogue unless the cytoplasmic calcium concentration is elevated by a separate signalling pathway, usually downstream of either glucose-dependent or sulfonylurea-dependent closure of K_ATP_ channels [13]. As well as stimulating insulin secretion, GLP1R agonists suppress glucagon release, slow gastric emptying, reduce appetite and improve myocardial performance during ischaemia [14, 15]. In rodent and in vitro studies, GLP-1 exhibits proliferative and anti-apoptotic effects on beta cells that could potentially be exploited to enhance beta cell mass, but the translatability of these findings to humans remains uncertain [16]. Some studies have also suggested effects of GLP-1 on liver metabolism [17] and memory/cognition [18], although the tissue localisation of the receptors underlying these effects is unclear [19]. Identifying the tissue distribution of GLP1R and providing mechanistic explanations for the wide variety of effects of GLP-1 will be critical for exploiting this receptor fully.

## GPCRs in the spotlight

The global success of GLP1R-based therapeutics has highlighted the benefits of targeting the gut–pancreatic axis, as well as the potential effectiveness of drugs that activate G_αs_-coupled receptors in pancreatic beta cells. Similar to the action of GLP-1, effects of other GPCR agonists also seem to exhibit glucose-dependence at the level of the beta cell. For example, GIP enhances glucose-dependent insulin secretion, likely because GIPR, like GLP1R, is coupled to cAMP elevation and requires a concomitant calcium signal before secretion is initiated. GIP-based therapeutics were not developed alongside GLP-1 in the 1990s because early studies concluded that the peptide loses effectiveness in the diabetic state, perhaps secondary to receptor downregulation in the beta cell [7]. There is, however, a growing interest in developing GIPR ligands in the form of new GLP-1–GIP dual agonist peptides [20]. This is just one example of a recent trend to develop peptides or peptide combinations that target two or three distinct GPCRs, each conferring a distinct metabolic profile. It has been proposed that this approach can potentially amplify beneficial metabolic effects whilst reducing the doses, and hence side effects, of individual peptide components [1]. For example, the combination of GLP-1 with glucagon, targets GLP1Rs and glucagon receptors (GCGRs) on the beta cell (both G_αs_-coupled), and in animal studies causes potent weight loss, perhaps by enhancing energy expenditure (via GCGR), whilst concomitantly reducing food intake (via GLP1R) [5].

GPR119 is a G_αs_-coupled receptor responsive to the natural ligands oleoylethanolamide and monooleoylglycerol [21]. A number of small molecule GPR119 agonists have been developed, aiming to enhance insulin secretion and reduce appetite by targeting gut endocrine cells alongside pancreatic beta cells. However, despite good evidence of efficacy in animal models, GPR119 agonists did not have metabolic benefits in humans with type 2 diabetes [2]. Why this should be the case remains unclear, and the possibility exists that the rodent and human enteroendocrine axes differ substantially in their use of this receptor. The findings with GPR119 raise another important question: how much endogenous GLP-1 will need to be secreted in response to L cell-targeted GPCR ligands in order to trigger a therapeutically relevant metabolic effect? Establishing the answer to this question is critical. It is not difficult to argue that the supraphysiological levels of GLP1R ligand achieved by GLP-1 mimetic therapy, or the approximately tenfold elevated GLP-1 and peptide YY levels found after gastric bypass surgery [22], have profound effects on appetite and metabolism. In comparison, attempts to target gut endocrine cells have produced relatively modest increases in plasma GLP-1 concentrations. Developing strategies that produce more substantially enhanced secretion of GLP-1 would seem an essential ambition.

Interestingly, glucose-dependent stimulation of insulin secretion is not restricted to G_αs_-coupled receptors, and has also been observed with G_αq_-coupled receptors linked to protein kinase C activation and inositol 1,4,5-trisphosphate (IP_3_)-dependent calcium release from intracellular stores. One of the classical G_αq_-coupled receptors linked to insulin secretion is the muscarinic acetylcholine receptor M_3_, responsible for the vagal stimulation of insulin release in some species [23]. Whereas M_3_ receptors are expressed in a wide variety of tissues, precluding their selection as drug targets for diabetes, FFAR1 (GPR40) and FFAR4 (GPR120) are G_αq_-coupled long-chain fatty acid receptors that have been a focus for small molecule development [24]. GPR40 is expressed in both pancreatic beta cells and gut endocrine cells, suggesting that its agonism might enhance insulin and GLP-1 secretion in type 2 diabetes. One of the GPR40 agonists to have progressed furthest in development, TAK-875, exhibited blood glucose lowering efficacy in Phase 3 clinical trials, but its clinical development was terminated because of concerns about liver toxicity [3]. Whether this was a class effect or a compound-specific off-target effect remains to be fully established, although reports that GPR40 is not expressed in hepatocytes and that TAK-875 impaired the function of bile acid-related transporters lends hope to the idea that structurally unrelated GPR40 ligands might not similarly target the liver [3, 25]. Indeed, newer GPR40 ligands have been developed that target different binding sites on the receptor and achieve ‘superagonism’ at the level of GLP-1 secretion [26].

The development of GPR120 agonists has lagged behind that of GPR40 ligands. This alternative NEFA receptor is expressed and functional in gut endocrine cells and linked to the release of GLP-1 and GIP [27, 28], but recent data suggest its pancreatic expression is high in somatostatin-producing delta cells and relatively low in beta cells [29]. Indeed, the application of flow cytometry techniques, transgenic mouse models with fluorescently labelled endocrine cell types and RNA sequencing has enabled the separation and transcriptomic analysis of different murine and human pancreatic islet cell types, providing molecular explanations for the well-recognised ability of certain drugs and physiological stimuli differentially to trigger or inhibit the release of one or more islet or gut hormones. Interestingly, GPR120 coupling to G proteins appears to be specific to the cell context: whereas G_αq_-dependent pathways are likely to underlie GPR120-dependent GLP-1 secretion [27], predominant G_αi_ coupling has been found in gastric ghrelin-secreting cells [30] and pancreatic delta cells [29].

It is physiologically and therapeutically relevant to note that whereas GLP-1 stimulates insulin secretion but suppresses glucagon release, GIP results in elevated circulating levels of both hormones [31, 32]. The suppression of plasma glucagon is one reason why GLP-1-based therapies are thought to be particularly effective in type 2 diabetes, although the mechanism underlying the inhibition of pancreatic alpha cells remains under debate. One proposed explanation is that a lower density of GLP1Rs than GIPRs in alpha cells results in a relatively modest elevation of cAMP in response to GLP-1 compared with GIP, and that this translates into opposite effects on glucagon release [33]. In perfused rat pancreas experiments, both GLP-1 and GIP stimulated insulin release, whereas GLP-1 decreased but GIP increased glucagon release, replicating observations made in humans [34]. In parallel, these studies revealed that both GLP-1 and GIP triggered somatostatin secretion and that the inhibitory effect of GLP-1 on glucagon release was lost in the presence of somatostatin receptor inhibitors. The data therefore suggest that one mechanism by which GLP-1 suppresses glucagon secretion is via the local release of somatostatin, consistent with the finding that *Glp1r* is expressed in only ∼10% of alpha cells [19].

## Exploring new GPCR targets for diabetes and obesity

Based on the extensive clinical experience of enhancing GLP1R activation for the treatment of diabetes, it can reasonably be concluded that highly desirable properties for new GPCR agonists would include: G_αs_- or G_αq_-coupled receptor activity on pancreatic beta and delta cells, low receptor activity on alpha cells, high activity on GLP-1-producing L cells and direct central nervous system effects to reduce food intake. Transcriptomic and functional comparisons between the different endocrine cell types, together with the recent identification of the human GPCR-ome [35] may yet yield some new GPCRs that fulfil these criteria which could be developed as candidate drug targets. However, many questions remain unanswered, perhaps the most notable of which is how to achieve substantial stimulation of postprandial gut endocrine secretion. Gut hormones are released physiologically by a variety of components of ingested food, which already recruit a host of GPCRs in enteroendocrine cells. If enteroendocrine cell GPCRs are already maximally activated by ingested food, then we will need to be somewhat cleverer if we are going to enhance gut hormone secretion further. A strategy that sensitises endocrine cells along the length of the gut to the arrival of food in the upper gastrointestinal tract might be effective, but how to achieve this ambition remains an enigma.

## Abbreviations

FFAR: Free fatty acid receptor
GIP: Glucose-dependent insulinotropic polypeptide
GIPR: Glucose-dependent insulinotropic polypeptide receptor
GLP-1: Glucagon-like peptide 1
GLP1R: Glucagon-like peptide 1 receptor
GPBAR1: G protein-coupled bile acid receptor
GPCR: G protein-coupled receptor
